# COMPLEMENT CONTRIBUTES TO ALZHEIMER’S DISEASE-INDUCED SYNAPSE DECLINE IN THE MURINE RETINA

**DOI:** 10.1093/geroni/igz038.3506

**Published:** 2019-11-08

**Authors:** Fenge Li, Danye Jiang, Melanie Samuel

**Affiliations:** Baylor College of Medicine, Houston, Texas, United States

## Abstract

Alzheimer's disease (AD) is among the most debilitating form of cognitive impairment in aged patients. Synapse deficits are thought to be a central trigger of neural miswiring and brain dysfunction in AD. However, the pathways that control synapse connectivity remain largely unknown. The retina is an easily accessible system with two distinct synapse layers and three cellular layers comprised of distinct neural types. In this study, we leveraged this system to assess synapse and cell integrity in the APPNLGF amyloid-beta AD mouse model. We showed that the expression of the complement component C3 is significantly increased in APPNLGF retina synapses, and that there is a significant decline of several synapse-associated markers by RT-PCR. These mice also display disorganized horizontal cell processes and visual function deficits. These results suggest that complement may drive AD-related changes in the synaptic and functional properties of the retina, which could serve as assessable preclinical biomarkers for AD. In ongoing studies, we are testing whether and how complement regulates synapse refinement and shapes retina synapse specificity in AD.

